# Spread of porcine circovirus associated disease (PCVAD) in Ontario (Canada) swine herds: Part II. Matched case-control study

**DOI:** 10.1186/1746-6148-6-58

**Published:** 2010-12-29

**Authors:** Zvonimir Poljak, Catherine E Dewey, Thomas Rosendal, Robert M Friendship, Beth Young, Olaf Berke

**Affiliations:** 1 Department of Population Medicine, University of Guelph, 50 Stone Road East, Guelph, Ontario, N1G 2W1, Canada; 2 University of Missouri, 900 East Campus Drive, Columbia, Missouri, USA, 65211

## Abstract

**Background:**

The emergence of porcine circovirus associated disease (PCVAD) was associated with high mortality in swine populations worldwide. Studies performed in different regions identified spatial, temporal, and spatio-temporal trends as factors contributing to patterns of the disease spread. Patterns consistent with spatial trend and spatio-temporal clustering were already identified in this dataset. On the basis of these results, we have further investigated the nature of local spread in this report. The primary objective of this study was to evaluate risk factors for incidence cases of reported PCVAD.

**Results:**

A time-matched case-control study was used as a study design approach, and conditional logistic regression as the analytical method. The main exposure of interest was local spread, which was defined as an unidentified mechanism of PCVAD spread between premises located within 3 kilometers of the Euclidean distance. Various modifications of variables indicative of local spread were also evaluated. The dataset contained 278 swine herds from Ontario originally sampled either from diagnostic laboratory submissions or directly from the target population. A PCVAD case was defined on the basis of the producer's recall. Existence of apparent local spread over the entire study period was confirmed (OR = 2.26, 95% CI: 1.06, 4.83), and was further identified to be time-varying in nature - herds experiencing outbreaks in the later part of the epidemic were more likely than control herds to be exposed to neighboring herds experiencing recent PCVAD outbreaks. More importantly, the pattern of local spread was driven by concurrent occurrence of PCVAD on premises under the same ownership (OR_EXACTwithin ownership _= 25.6, 95% CI: 3.4, +inf; OR_EXACToutside ownership _= 1.3, 95% CI: 0.45, 3.3). Other significant factors included PRRSv status of a herd (OR_EXACT _= 1.9, 95% CI: 1.0, 3.9), after adjusting for geographical location by including the binary effect of the easting coordinate (Easting > 600 km = 1; OR_EXACT _= 1.8, 95% CI: 0.5, 5.6).

**Conclusions:**

These results preclude any conclusion regarding the existence of a mechanism of local spread through airborne transmission or indirectly through contaminated fomites or vectors, as simultaneous emergence of PCVAD could also be a result of concurrent change in contributing factors due to other mechanisms within ownerships.

## Background

Porcine circovirus associated disease (PCVAD), also known as porcine circovirus disease and previously as post weaning multisystemic wasting syndrome (PMWS), emerged in the early 1990's and soon became a major animal health problem in many swine-producing regions worldwide [1,2]. The disease has several clinical presentations and can result in high mortality in growing pigs. Most frequently, PCVAD clinically manifests as excessive and rapid weight loss in growing pigs, respiratory illness, and increased mortality. Post-mortem findings frequently include generalized lymphadenopathy on macroscopic examination, and lymphoid depletion and/or histiocytic replacement of follicles in lymphoid tissues on microscopic examination [3,4]. Porcine circovirus type 2 (PCV2) has been recognized as a necessary cause of PCVAD, but several other infectious and non-infectious factors have been identified as component causes acting through various mechanisms [4,5]. Studies of epidemics at the herd-level within distinct geographical regions [6-10] are disproportionately rare, relative to the impact that PCVAD had on production, possibly due to diagnostic uncertainty and availability of geographical and temporal information required to make inferences about the spread. Reports describing large-scale extensive investigations of PCVAD involving trace-back and trace-out are limited [6,10], likely reflecting the epidemiology of PCV2 infection, and the non-reportable nature of the disease. Management changes [11,12] and vaccination have been used to control the disease. In North America, commercial vaccines first became available during 2006, and since then became one of the most commonly used vaccines in growing pigs.

Studies conducted in different regions before vaccine introduction identified spatial, temporal, and spatio-temporal trends as factors contributing to patterns of the disease spread [8,9], although not under all conditions. In our accompanying article [13], we have identified spatial trends in PCVAD risk and described the nature of spatio-temporal dependence. This dependence was interpreted as the existence of a pattern of local spread. On the basis of this exploratory analysis, we have postulated further hypotheses about local spread and investigated them here. The primary objective of this study was to evaluate risk factors for incidence cases of reported PCVAD. The main exposure of interest was local spread, which in this manuscript was defined as the unidentified mechanism of PCVAD spread from infectious to susceptible premises located within 3 km of the Euclidean distance.

## Results

Table 1 contains descriptive statistics for a subset of variables that were included in an inferential analysis based on the time of PCVAD outbreaks (in case herds) and on time of selection as a time-matched control (in the applicable risk set). We will refer to the sampling times as herds, although they reflect the status of a herd at a particular time (month) since December of 2003. The high proportion of PRRSv-positive herds in each PCVAD status is a reflection of the sampling strategy that favored selection of PRRSv-positive herds. Of note is that the proportion of herds that had "high risk local exposure" (HRLE) at the time of sampling was relatively low for case and control herds. Aside from being a characteristic of the study population, this might be a characteristic of the target population as well, because pork producers in Ontario generally try to maintain distance between swine premises for biosecurity purposes. In addition, it is noteworthy that control herds did not have exposure to PCVAD-infectious herds within ownership, unlike the case herds. In the case group, a higher proportion of local exposure was from PCVAD-infectious premises that were in different ownership than from PCVAD-infectious herds within the same ownership (Table 1, 4.7% versus 2.9%, respectively).

**Table 1:** Descriptive statistics of major demographic variables of observed and the matched dataset

	Control times					Case times				
		
Variable	Mean	Median	sd*	iqr*	N*	Mean	Median	sd*	iqr*	N*
Herd PRRSv status (proportion positive)	0.86	1.00	0.35	0.00	510	0.91	1.00	0.29	0.00	170
Cumulative local exposure (proportion)	0.16		0.37		510	0.17		0.38		170
Number of high-risk exposures	0.04	0.00	0.20	0.00	510	0.08	0.00	0.30	0.00	170
High-risk local exposure (proportion)	0.04		0.19		510	0.08		0.27		170
Number of nursery contacts/month	1.09	0.00	2.41	0.50	510	0.75	0.00	2.09	0.00	170
Number of feed contacts/month	4.06	4.00	2.53	2.33	510	4.59	4.00	3.73	4.00	170
Number of direct contacts^a^/month	1.39	0.00	2.46	2.00	510	1.13	0.00	2.20	1.17	170
Number of indirect contacts^b^/month	6.61	5.67	4.25	3.67	510	7.01	6.00	4.08	4.00	170
Total capacity for growing animals	2161.33	2000.00	1586.11	1525.00	510	2180.14	2000.00	1461.31	1520.00	170
Easting (100 kms)	4.87	4.87	0.57	0.65	510	5.05	5.01	0.71	0.50	170
Number of sows	321.60	240.00	281.66	280.00	279	357.13	240.00	398.05	290.00	92
Number of nursery pigs	1363.81	1000.00	1170.90	1600.00	396	1355.88	900.00	1400.92	1600.00	116
Number of finisher pigs	1308.81	1100.00	1083.18	361.00	510	1421.15	1250.00	982.14	1350.00	127
Number of breeding contacts/month	5.11	4.08	4.27	6.92	278	5.57	4.67	4.36	7.38	92
		
Herd type: farrow-to-finish	43.14				220	41.18				70
Herd type: farrow-to-grow	11.37				58	12.94				22
Herd type: finisher	21.76				111	30.00				51
Herd type: nursery	17.84				91	12.35				21
Herd type: nursery and finisher	5.88				30	3.53				6
		
High risk exposure: not present	96.27				491	92.35				157
High risk exposure: within ownership	0.00				0	2.94				5
High risk exposure: outside ownership	3.73				19	4.71				8

*sd = standard deviation, iqr = interquartile range, N = number of observations

^a ^frequency of monthly contacts through incoming movement of nursery and finisher pigs

^b ^frequency of monthly contacts through incoming visits of service vehicles without pigs (feed deliveries, veterinarians, veterinary suppliers, other service personnel)

Table 2 details measures of univariable associations obtained from conditional logistic regression based on assumption of Gaussian distribution (GD) for the test statistic. Additionally, measures of univariable associations obtained from the exact logistic regression were obtained for nominal variables (Table 2). The exact method provided more reasonable point estimates and test of significance once ownership similarity between case status and HRLE was taken into account. This risk factor appeared to follow a "dose-response" pattern because the odds increased as the number of infectious herds in the 3-km zone increased (Table 2, LNHRH). Local spread seemed to be driven primarily by high-risk local exposure to infectious herds within the ownership (Table 2; OWHRLE). In addition to variables that met the criteria for inclusion into multivariable analysis, Table 2 also contains some non-significant associations of interest, such as cumulative local exposure (CLE), number of direct contacts/month, number of indirect contacts, and number of breeding contacts/month for farms where the breeding part of the swine operation was present. Although not shown in Table 2, non-significant associations were also detected for frequency of direct contacts/month through semen, gilts and boars, growing pig shipments, veterinary visits, veterinary supplies visits, and rodent control visits. A variable depicting frequency of feed delivery (Table 2, number of feed contacts/month) was not statistically significant (*P *> 0.10) once herds with frequency of feed contacts of >25/month were excluded from the analysis (n = 4, n strata = 1). Similarly, a candidate final model that contained frequency of visits by the feed suppliers also indicated that the association was not statistically significant once this influential point was removed from analysis. On this basis, a model containing frequency of feed contacts was not considered a reasonable candidate for the final model. Interactions between local high-risk exposure and herd PRRSv status (Likelihood ratio test [LRT], *P *= 0.25), or the linear effect of easting (LRT, *P *= 0.20), or between linear effect of easting and herd PRRS status (*P *= 0.65) were all not significant. Interaction between time (measured as number of months between December of 2003 and the date of matching) and HRLE was the only statistically significant interaction term of time (LRT, *P *= 0.01). This interaction suggested that HRLE was numerically protective in January of 2004 (Time = 1, *P *= 0.09, OR = 0.04, 95% CI: 0.0, 1.7), reversed into a risk factor (based on point estimate only) in May of 2005 (Time = 17, *P *= 0.97, OR = 1.0, 95% CI: 0.3, 3.1) and a statistically significant risk factor in October of 2005 (Time = 21, *P *= 0.02, OR = 2.7, CI: 1.1, 6.4), and remained a statistically significant risk factor in December of 2005 (Time = 24, *P *< 0.01, OR = 4.0, 95% CI: 1.5, 10.9), December of 2006 (Time = 36, *P *< 0.01, OR = 42.0, 95% CI: 2.6, 673.1), and October of 2007 when the last outbreak in this dataset was reported (Time = 46, *P *= 0.01, OR = ~270, 95% CI: 3.1, ~22,900).

**Table 2:** Univariable associations for variables considered for multivariable analysis, including interaction between high-risk local exposure and time of matching

Variable	Odds ratio	95% CI	***P***^**a**^	**Overall *P***^**b**^
*Estimates based on the maximum likelihood method using assumption of Gaussian distribution*				

Herd PRRSv-positive status (no = ref^c^)	1.56	(0.89, 2.75)	0.121	0.107
Herd type				0.090
Farrow-to-finish	- -	-	-	
Farrow-to-grow	1.23	(0.70, 2.18)	0.473	
Finisher	1.47	(0.96, 2.26)	0.077	
Nursery	0.74	(0.43, 1.27)	0.275	
Nursery and finisher	0.59	(0.23, 1.51)	0.274	
Easting (100 kms)				0.002
linear	1.54	(1.17, 2.03)	0.002	
Cumulative local exposure (CLE) (no = ref)	1.05	(0.64, 1.70)	0.852	0.852
High-risk local exposure(HRLE) (no = ref)	2.26	(1.06, 4.83)	0.035	0.040
HRLE*Time interaction model				0.005
HRLE	0.04	(0.00, 1.74)	0.094	-
HRLE*Time (Month)	1.21	(1.02, 1.44)	0.031	0.0128
Local # of high-risk herds LNHRH (continuous)	2.16	(1.06, 4.39)	0.033	0.036
Local # of high-risk herds LNHRH (nominal; 0 = ref)				0.111
One	2.20	(1.02, 4.76)	0.045	
Two	4.03	(0.24, 67.50)	0.333	
Ownership-adjusted high-risk local exposure (OWHRLE; No high-risk local exposure = ref)				0.001
High-risk local exposure within ownership	1.0E+7	(not estimated)	0.984	
High-risk local exposure outside of ownership	1.31	(0.54, 3.17)	0.555	
Number of direct contacts^d^/month	0.95	(0.88, 1.03)	0.221	0.205
Number of indirect contacts/month	1.02	(0.98, 1.07)	0.256	0.263
Number of nursery contacts/month	0.93	(0.85, 1.02)	0.106	0.086
Number of breeding contacts/month^e^	1.03	(0.96, 1.10)	0.407	0.407
Number of feed contacts/month	1.06	(1.00, 1.12)	0.043	0.045

*Estimates of the high-risk local exposure obtained using exact logistic regression*				

Herd PRRSv-positive status (no = ref)	1.56	(0.87, 2.95)	0.151	0.151
Cumulative local exposure (CLE) (no = ref)	1.05	(0.62, 1.74)	0.901	0.901
High-risk local exposure(HRLE) (no = ref)	2.26	(0.97, 5.18)	0.049	0.049
Local # of high-risk herds LNHRH (continuous)	2.16	(0.99, 4.69)	0.043	0.043
Local # of high-risk herds LNHRH (nominal; 0 = ref)				0.108
One	2.18	(0.92, 5.04)	0.049	
Two	4.03	(0.05, 330.32)	0.367	
Ownership-adjusted high-risk local exposure (OWHRLE; No high-risk local exposure = ref)				0.001
High-risk local exposure within ownership	20.17^f^	(2.75, +inf)	0.001	
High-risk local exposure outside of ownership	1.31	(0.46, 3.39)	0.634	

^a ^Based on the Wald test for conditional maximum likelihood.

^b ^Based on the Likelihood ratio test for conditional maximum likelihood.

^c ^ref = referent group.

^d ^Frequency of all contacts was calculated on the basis of visits to herds.

^e ^Based on 230 observations where such information was applicable.

^f ^Median unbiased estimator.

Table 3 lists the final multivariable model fitted using the likelihood method and the exact method. The exact logistic regression contained only the variables linked with local spread as exposure of primary interest, for which coefficients were provided, and herd PRRSv status and binary variable of easting (>600 km East) as confounders. A final exact logistic regression model including easting as a continuous explanatory variable could not be fit.

**Table 3:** Final multivariable model obtained using assumptions of the Gaussian distribution or exact method

Variable	Odds Ratio	95% Conf. Interval	***P***^**a**^	**Overall *P***^**b**^
*Final model obtained based on the maximum likelihood method using assumption of Gaussian distribution*				
Ownership-adjusted high-risk local exposure (OWHRLE; No high-risk local exposure = ref)				<0.001
High-risk local exposure within ownership	8.4E+6	(not estimated)	0.982	
High-risk local exposure outside of ownership	1.32	(0.54, 3.22)	0.542	
Easting (100 kms)	1.65	(1.22, 2.21)	0.001	<0.001
Herd PRRSv status (positive)	2.25	(1.18, 4.28)	0.013	0.008
N obs = 680; n groups = 170, LR chisq = 29.4, df = 4, *P *< 0.001, AIC = 450				

*Adjusted estimates of the high-risk local exposure obtained using exact logistic regression *^c^				

Ownership-adjusted high-risk local exposure (OWHRLE; No high-risk local exposure = ref)				<0.001
High-risk local exposure within ownership	^d ^25.65	(3.40, +Inf)	<0.001	
High-risk local exposure outside of ownership	1.27	(0.45, 3.30)	0.640	
Easting binary (>600 km east = 1)	1.81	(0.53, 5.62)	0.378	0.378
Herd PRRSv status (positive)	1.93	(1.02, 3.91)	0.04	0.04
N obs = 680; n groups = 170, Model score = 19.98, *P *< 0.001				

^a ^*P*-values are based on the Wald test for estimates of a model obtained using maximum likelihood method and based on Score test for estimates obtained using exact method.

^b ^Overall *P*-values are based on the likelihood ratio test for estimates of a model obtained using maximum likelihood method and based on Score test for estimates obtained using exact method.

^c ^Estimates of the local spread where obtained from the exact logistic regression after adjusting for the herd PRRS status and easting as a binary variable. Adjustment for the easting as a continuous explanatory variable could not be evaluated.

^d ^Best median unbiased estimates.

## Discussion

In the accompanying article [13], spatio-temporal clustering at the small spatial and temporal scale was detected, suggesting that local spread was a possible pattern of PCVAD spread. Specifically, using the space-time K-function, we detected considerable spatio-temporal clustering of cases up to approximately 4.5 km of spatial and 5 mo of temporal distance to a PCVAD case (proportional increase of >100% of what was expected), with the highest increase at 2 km of spatial and 1 mo of temporal distance. In contrast to the previous method, the approach taken here required that we define local exposure using fixed spatial and temporal distance, recognizing that this could lead to some misclassification of exposure. We aimed to use cutpoints that would provide a balance between specificity of defining exposures (higher if shorter distance used) and number of herds that would be classified as exposed (higher if longer distance used). Thus, three km of spatial distance was selected subjectively and used as the only analytical approach for geographical distance, while a temporal distance of 3 mo was used as the most important, but not the only, temporal cutpoint. This spatial cutpoint was in concordance with how local spread was defined in a field during the 2001 outbreak of foot-and-mouth disease in the UK [14]. Our assumption was that the causative agent of PCVAD from the initially infected premises would not be more contagious to neighboring farms than the foot-and-mouth disease virus.

The limitation of our original finding of the existence of apparent local spread was that the results were not adjusted for factors that could facilitate such a conclusion, even in the complete absence of radial spread of a causative agent from an infected farm to a neighboring susceptible herd. One such factor is ownership of the premises. In at least some Ontario regions, premises under the same ownership cluster geographically, and finding a pattern of spatial or spatio-temporal clustering of infectious disease might be facilitated by introducing infected animals from the same source, concurrent change in management, or frequency of other component causes, or simply by recall bias of the interviewee. It was therefore of interest to explore the nature of local spread after adjusting for ownership, and a time-matched case-control study was considered a suitable design for this objective.

Of interest for this investigation was finding that CLE was not associated with the emergence of incident PCVAD cases. Thus, even if local spread existed, the farms that reported a "historical" PCVAD problem did not contribute to the emergence of incident PCVAD cases in the neighborhood, at least in this study. On the other hand, the existence of spatio-temporal clustering at the small geographical and temporal scale - described in the accompanying article [13] - was confirmed in this study by having a significant variable that was indicative of HRLE. The results suggested that PCVAD-positive herds were more likely to have neighboring herds (up to 3 km) involved in a recent PCVAD outbreak (0 to 2 mo prior, total = 3 mo), than the PCVAD-negative herds. It is also noteworthy that this local exposure showed some limited dose-response relationship, with odds of being a case higher for herds that had two infectious herds within a 3-km distance than for herds with no infectious herds or only one such herd. Interaction of HRLE with time was biologically plausible and in agreement with previously reported results of PCVAD spread. Woodbine et al reported that radial spread was important in the later period of PCVAD spread in Great Britain [8,9]. When considered as the only term in the model, local spread has a time-varying nature (statistical interaction with time). The variable indicative of local spread (HRLE) became statistically significant in October of 2005, corresponding approximately to the 50^th ^percentile of reported PCVAD outbreak times, and remained significant until the end of the study.

However, when HRLE was partitioned into within-ownership and outside-ownership, it was obvious that the likelihood of local spread was determined primarily by the co-existence of neighboring herds under the same ownership that experienced a PCVAD outbreak at concurrent times. The point estimate for the odds ratio of "High-risk local exposure within ownership" in the final model was very high and with very wide confidence intervals. This certainly is a limitation of this analysis and is a consequence of absence of this exposure in the control herds, as visible from descriptive statistics. However, collectively these results suggest that it is possible that such a mechanism of "local spread" could be driven by mechanisms that do not involve any type of spatial interaction between the neighboring farms (e.g., introduction from a common source, simultaneous change in management or prevalence of other component causes, or recall bias). We have no information that would allow us to further investigate these hypotheses. Our results, however, suggest that exploratory spatial and spatio-temporal analysis should be substantiated by additional analyses that will take ownership or pig movement into consideration, particularly in zones where spatial clustering of premises under the same ownership exists. We defined ownership at the highest possible level, and this might have some impact on the results. Large systems might have had several independent pig flows, and, theoretically, neighboring premises might belong to different flows (have different pig sources). In such situations, the nature of high-risk exposure would likely be incorrectly classified as within-ownership. We believe that the latter scenario is relatively infrequent and would likely have a low impact on overall results and general interpretation. This issue of ownership, however, does warrant access to suitable ownership and movement databases that would allow epidemiologists to adjust their analysis for the proper ownership and the contact structure. In addition it is important to note that the interaction of HRLE with time was not significant once the ownership was accounted for. Although the interaction of HRLE with time was a biologically plausible term to include in the final model, the OWHRLE was selected instead because we considered these results were more insightful in explaining the underlying biological processes.

The final model also contained two additional confounders: herd PRRS status and the linear effect of easting. PRRS infection in individual pigs has been identified as a factor contributing to PCVAD due to its effect on the immune system, and as a risk factor for PCVAD expression in observational studies [15-17].

Also of interest was the finding that the frequency of direct contacts, including through breeding animals for a subset of suitable herds, was not identified as a risk factor. This was a surprising finding because introduction of infectious agents through live animals is considered an important component of PCVAD emergence [8,9,12]. Two factors might have contributed to this. First, the frequency of visits used as a covariate in this analysis does not necessarily correspond to the time prior to a PCVAD outbreak in a herd. It is possible that the frequency of direct contacts changed in response to the PCVAD outbreak or an outbreak of another disease (e.g., PRRS). While short-term change in frequency of movements can be expected as a part of outbreak control, a long-term change is much less likely because it would require a change in the type of farming system, typically associated with high cost. The second possible explanation is that emergence of PCVAD is linked with the specific PCV2 source, and not with the general measure of frequency of incoming visits or movements. This is of note because either PCV2 as a necessary cause, or PRRSv or other agents as component causes, could spread through movement of animals and have an impact on disease emergence.

Although frequency of feed deliveries was identified as the only significant factor related to visits, this was most likely a chance finding, on the basis of the following rationale. The significance of this association was influenced by one herd that reported a very high frequency of feed deliveries, but the feed was delivered from the farm's own feed mill. This would contrast with the logical explanation that a causative agent was distributed either through feed from an external point source, or indirectly through delivery trucks between farms. Feed has been discussed as a possible source previously [12]. Evaluation of PCV2 transmission through feed containing contaminated porcine plasma yielded opposing results under experimental conditions [18,19].

Case definition was the largest limitation of this study, and the rationale for including it in the analysis is given in the accompanying article [13]. Briefly, preliminary analysis with more specific case definitions gave us similar results. Additionally, the evaluation of epidemic spread under current conditions using complete set of diagnostic criteria is not possible because of high vaccination usage and endemic presence of PCV-2 infection in many herds. Introduction of commercial PCV2 vaccines likely influenced the development of this outbreak. A major supplier gradually introduced its product to Canada in April of 2006 [20,21]. We fitted the final model only with cases that reported PCVAD prior to April of 2006 and found results (not reported) similar to our final model. We thus believe that results reported here were not influenced in a substantial manner by vaccination.

## Conclusions

In conclusion, the spread of PCVAD due to high-risk local exposure was primarily driven by herd ownership. It was therefore impossible to distinguish local spread from common direct or indirect sources that contributed to the emergence of disease concurrently in different premises under the same ownership. Surprisingly, frequency of direct or indirect contacts did not differ between case and control herds. This also applies to frequency of feed delivery, which, although statistically significant, was driven by a single highly influential point, which in itself did not align with a biological rationale for spread through feed deliveries. Two risk factors that remained stable were herd PRRSv status and directional spread in a western direction. Multivariable analysis following exploratory spatial analyses proved to be a useful complement for this study. After completion of this analysis, our conclusions about disease spread have changed. Accounting for ownership and contact structure will become increasingly important in swine populations because investigation of spatial and temporal information alone would have yielded incomplete results and different recommendations.

## Methods

### Dataset

The full description of the data sources and data management steps is provided in the accompanying article [13]. Briefly, 278 herds located in southern Ontario were included in this study initially using two different sampling mechanisms: (i) sampling of herds positive for porcine reproductive and respiratory syndrome virus (PRRSv) using data from a diagnostic laboratory (Animal Health Laboratory, University of Guelph, Guelph, ON, Canada), based on rtPCR-positive diagnostic findings, and (ii) sampling of PRRSv-negative herds, based on assessment of a herd veterinarian. Herds were classified into PCVAD-positive and negative herds on the basis of producers' assessments, and results obtained during exploratory spatial analysis were used to guide the design of this case-control study.

### Exposures of interest

#### Local exposure

Estimation of the spatio-temporal K-function suggested significant spatio-temporal clustering over small spatial and temporal distance [13]. The risk was proportionally higher (relative increase >1) for up to ~ 4.5 km of spatial and up to ~5 mo of temporal distance from an arbitrary herd experiencing a PCVAD outbreak, and was increasing as both distances (in Euclidean distance and in time) to an arbitrary selected PCVAD-positive herd decreased. This result led us to generate a binary variable depicting "high-risk local exposure" (HRLE) to a PCVAD herd, whereby a herd would be considered positive for HRLE if it was within 3 km of a spatial distance from a herd that started experiencing a PCVAD outbreak that month or up to 2 mo earlier (total of 3 mo). Maximum relevant spatial and temporal distance was partly based on the spatio-temporal K-function, while considering that a shorter distance to the nearest herd could increase data scarcity and complicate analysis. A binary variable depicting HRLE to PCVAD was generated from available spatial and temporal data in the following way. In the first step, the herd-level dataset was expanded to include 52 mo duration of the period for each herd. This time period corresponds to the duration of time between December of 2003 (Month = 0), after which date producers started reporting outbreaks, and April of 2008 (Month = 52), when the last producer was interviewed. Each PCVAD-positive herd was considered infectious in the month when PCVAD was first reported and in the two subsequent months for a total duration of 3 mo, as non-infectious otherwise, and as censored after the month of the interview. In the second step, data for each month were exported to GIS software (ArcGIS 9.1) and buffering for each infectious herd was performed. Each buffer had a radius of 3 km and all herds that were contained within each buffer were selected and considered exposed to a high-risk PCVAD herd. In the third step, only herds exposed to another high-risk PCVAD herd were classified as exposed. This was done by removing herds that were used to generate buffers (infectious herds) using basic geoprocessing (**PBSMapping; R 2.8.0**) and data processing operations (**R 2.8.0**). Thus, a herd was considered positive for HRLE in a particular month of the study period only if it had another infectious PCVAD herd (herd was infectious over a period of 3 mo) within a 3-km distance. This operation of generating binary variable HRLE was repeated for each month, and the resulting dataset served to generate a new set of variables that investigated different aspects of local exposure.

The second variable of local exposure was generated by counting the number of herds that experienced an outbreak of PCVAD in the month of interest or the preceding 2 mo and were located within a 3-km radius around a herd of interest. We refer to this variable as "local number of high-risk herds" (LNHRH). The third variable of local exposure was generated by taking ownership of the exposing (infectious) herd and the exposed herd into account. A list of ownerships and contact names was available for each herd, where ownership was considered a membership in a particular production system at the highest possible level. If ownership was unknown, we used a contact name as a proxy for ownership. This list was used in order to depict whether an exposing (infectious) herd was under the same ownership as a herd that was exposed. This resulted in generation of a nominal variable, "ownership-adjusted high-risk local exposure" (OWHRLE) with three levels: (i) no high-risk local exposure (OWHRLEno), (ii) high-risk local exposure within ownership (OWHRLEin), and (iii) high-risk local exposure outside of ownership (OWHRLEout). In addition, a binary variable that depicted exposure of a herd to another herd that had experienced PCVAD at any point in the past (not only during the high-risk period) and that was ≤3 km distant was also created during data processing. We refer to this variable as "cumulative local exposure" (CLE).

#### Demographic variables

Other variables considered for analysis were already present in the dataset and were considered as time-invariant and explored in the accompanying article [13]: (i) PRRSv status, (ii) herd type, (iii) capacity for growing pigs on premises (nursery and finisher capacity). Additionally, monthly incoming contact rates from outside sources were calculated for: (i) frequency of monthly contacts through animal movement - direct contacts - and number of direct sources (animal movements of nursery and finisher pigs to premises), (ii) frequency of indirect monthly contacts (through feed shipments, veterinary visits, veterinary supplier visits, and service visits), (iii) number of indirect monthly contacts through each of the indirect source types, (iv) number of monthly contacts through breeding stock supply (gilts and boars) for sites that had breeding (sows) present on-site. An assumption was made while making this calculation that contact rates and other time-variant variables were unchanged since the time of the PCVAD outbreak. In cases where a variable had missing values, we imputed the values using routines available in commercial software (Stata 10), and using all other variables with available information. In the final dataset, values of four observations from two different herds were imputed (out of 680 observations in the matched dataset).

### Matching

Incidence density sampling was used for matching. Strata were constructed so that each PCVAD case herd was individually time-matched to three randomly selected control herds from the available risk set on the month of PCVAD occurrence in a case herd. The available risk set in a specific month were all herds that: (i) were not PCVAD-positive (infectious) in this month or earlier (current or previous case herds), (ii) were not interviewed prior to this month, and (iii) were not already matched as controls to another case in that specific month (simple random sampling without replacement). Herds could have been, however, selected as controls to another PCVAD-case in subsequent months. Exposure status with respect to HRLE, LNHRH, OWHRLE, and CLE, and other time-variant variables, was assigned to each case and control herd using the approach described.

### Statistical analysis

In the univariable analysis, conditional logistic regression based on Gaussian distribution was used to evaluate each factor of interest. Variables indicative of local exposure and other categorical variables were additionally tested using exact conditional logistic regression. Univariable models were first examined and variables that were either significant using a liberal *P *value of <.15, or were considered confounders in the target population, were included in the multivariable modeling. Although the main effect of time (matching variable) cannot be included as an explanatory variable in this analysis, the significance of interaction between time and each explanatory variable can be investigated [22]. Such interaction terms were therefore tested with each variable that, on its own, was significant at *P *< 0.15. A likelihood ratio test was used to test interactions with linear and quadratic effects of time. Testing was performed at this stage, instead of at the end of the multivariable analysis, because it was anticipated that the ratio of number of observations and strata per variable evaluated would be more desirable during this univariable stage. The choice of the final multivariable model was based partly on biological rationale and partly on statistical significance. A final model could not be fitted using exact conditional logistic regression. Therefore, a full model containing all variables was fitted using GD assumptions, and additionally, a "full model" with binary variable for easting (>600 km East) was fitted using exact conditional logistic regression. Since residual diagnostics were not available for conditional logistic regression, a model containing identical covariates as the final model in the conditional logistic regression was refitted using ordinary logistic regression and influence statistics and residuals were evaluated. Identification of any influential points prompted refitting the model using conditional logistic regression, omitting influential points and assessing the change in coefficients and their statistical significance. The exact *P*-value and the associated 95% confidence intervals were based on the score method. The best median unbiased estimates were used when point estimates could not be obtained for coefficients. Models were run using commercial software (Stata 10 SE, College Station, TX).

## Authors' contributions

ZP participated in study design, and data processing, analyzed data statistically, interpreted results, and wrote the initial draft of the manuscript. CED designed and obtained funding for the large monitoring study, participated in study design, developed the questionnaire, and commented on the initial draft. TR participated in study design, questionnaire development, data processing and commented on the initial draft. RMF participated in questionnaire development and commented on the initial draft. BY collected data, and provided comments on the initial draft. OB provided comments on the initial draft and statistical methodology. All authors read and approved the final version.
